# Prevalence of Premenstrual Syndrome Levels and Its Management Among Female Students of Medical and Non-Medical Colleges in Riyadh

**DOI:** 10.7759/cureus.11595

**Published:** 2020-11-20

**Authors:** Maryam Muhammad Ali Majeed-Saidan, Nourah AlKharraz, Kayan Kaaki, Noura AlTawil, Sara Alenezy, Shaik Shaffi Ahamed

**Affiliations:** 1 Family and Community Medicine, King Saud University, Riyadh, SAU; 2 Medicine, King Saud University, Riyadh, SAU; 3 Biostatistics, King Saud University, Riyadh, SAU

**Keywords:** medical students, saudi arabia, depression, premenstrual syndrome, stress, anxiety

## Abstract

Objective

The purpose of this study is to measure the prevalence of premenstrual syndrome (PMS), its management, and its possible association with depression, anxiety, and stress among female medical and non-medical students.

Methods

A cross-sectional study was conducted at King Khalid University Hospital and King Saud University Female Campus. A self-administered questionnaire following the American College of Obstetricians and Gynecologists (ACOG) criteria was used to measure levels of PMS. It also included the Depression Anxiety Stress Scale (DASS) to measure depression, anxiety, and stress. The inclusion criteria were: females of reproductive age who had menstrual cycles for two consecutive months. Meanwhile, the exclusion criteria included gynecological or psychological problems, pregnancy, and the use of oral contraceptive pills. Of the 513 responses, 414 met the above criteria and were used in the study. An analysis was done using the Statistical Package for the Social Sciences (SPSS, version. 21.0, IBM Armonk, NY). To compare the mean values of quantitative variables, the student’s t-test for independent samples was used. Accordingly, Pearson’s correlation quantified the linear relationship between the two quantitative variables.

Results

The majority of female students have a moderate level of PMS, and only 8% have severe PMS. Notably, 8.9% of the students have moderate anxiety while 1.7% and 0.7% have severe and extremely severe anxiety, respectively. Moreover, 11.8% and 3.4% of the students have moderate depression and stress, respectively, whereas 1.7% have severe depression. The results show a positive correlation between PMS and anxiety, depression, and stress. Depression and stress scores vary significantly between medical and non-medical students.

Conclusion

Premenstrual syndrome is a prevalent issue among females, and it can negatively affect their health. There is a need to study PMS thoroughly to optimize and guide its management for further improving women’s health.

## Introduction

Premenstrual syndrome (PMS) is defined as the presence of physical, behavioral, and mood symptoms that arise in the luteal phase of the menstrual cycle, resolve after menstruation, and have an effect on women’s daily life [1-2]. Another study defines PMS as reverent episodes of physical and psychological signs and symptoms that occur in the luteal phase; these disappear over the onset of periodic hemorrhage [3]. PMS is quite a prevalent issue among women all over the world and is yet underestimated. It varies in severity among women, ranging from mild to severe [3-4]. Ozlem et al. summed the prevalence of PMS from different Turkish studies to be between 5% and 79% [5]. Notably, PMS can negatively affect women’s lives, both physically and psychologically. A study at King Faisal University in Al Ahsa, Saudi Arabia found the levels of severity of PMS as follows: 45% mild, 32.6% moderate, and 22.4% severe [6]. The American College of Obstetrics and Gynecology (ACOG) requires one somatic and one affective symptom to diagnose PMS [7]. The presence of one affective symptom in the criteria led us to believe in the possible correlation between PMS and anxiety, depression, and stress. Since PMS is widely experienced in females [8], its management varies with different approaches to symptom relief, ranging from home remedies and hot drinks to prescription drugs [9-10], over-the-counter medication, and consultation with a gynecologist or psychiatrist.

As PMS is a poorly researched topic in our region, there is a deficit in the available pool of knowledge regarding its severity. Our study, therefore, aims to measure and compare the levels of PMS and to determine the association between the severity of PMS and depression, anxiety, and stress among female students of medical and non-medical colleges. Moreover, this study aims to quantify and compare the methods of PMS management to relieve its symptoms. To achieve these objectives, we asked participants to fill out a questionnaire containing both the ACOG and Depression Anxiety Stress Scale (DASS).

## Materials and methods

Study design and setting

This cross-sectional study took place during the period from December 2017 to May 2018. The study included medical students from different universities in Riyadh, including King Saud University, Princess Nourah University, Alfaisal University, and Alfarabi College. Non-medical students were chosen from the colleges of Linguistics, Business, Science, and Computer Science at King Saud University. The sample was predetermined to be 422 from each group, assuming 50% of medical and non-medical students suffer from PMS, with ±5% precision and a 0.05 level of significance; hence, the target was 231 medical and 231 non-medical female students. Anticipating 10% non-response, 38 samples were added to each section.

Recruitment of participants

Due to the limited number of female medical students, all of them were included in the study while non-medical students were chosen through a systematic random sampling technique. The study included females of reproductive age (i.e., between 12 and 51) and have had their menstrual cycle for the past two months. Students who did not have their menstrual cycle for the past two consecutive months or have been diagnosed with any medical, gynecological, or psychosis were excluded from the study. Participants who were pregnant or using oral contraceptive pills or other drugs that may influence the period were also excluded. 

Study procedures

The data were collected through a self-administered survey that contained sociodemographic information, the ACOG criteria, methods of PMS management, and the DASS criteria. The surveys were sent to the participants via email in both Arabic and English to suit their preferences. Sociodemographic data included the participants’ age, weight, height, nationality, major, current marital status, and length of menstruation.

Development of the questionnaire 

We used the ACOG PMS diagnostic criteria that include both somatic (e.g., change in thirst or appetite, breast tenderness) and behavioral symptoms (e.g., anger, unhappiness). Having at least one somatic and one behavioral symptom qualify for a diagnosis of PMS. In the ACOG criteria, each symptom is rated on a four-point Likert scale, ranging from 0 = “Did not apply to me at all” to 3 = “Applied to me very much or most of the time.” The participants were then classified as mild, moderate, or severe after the total score of PMS symptoms was calculated and then divided by the number of symptoms experienced. Meanwhile, the DASS questionnaire includes 21 entities that measure the negative emotional states of depression, anxiety, and stress on a scale of 0 to 3. The sum of the symptom scores was calculated to classify the participants into the three categories.

Statistical analysis

The data were analyzed using the Statistical Package for the Social Sciences (SPSS, version. 21.0, IBM Armonk, NY). Descriptive statistics (i.e., mean, standard deviation, frequencies, percentages) were used to describe the study’s outcome variables. Pearson’s correlation was used to quantify the linear relationship between two related quantitative variables. Student’s t-test for independent samples was used to compare the mean values of quantitative variables between medical and non-medical students. A p-value of < = 0.05 was used to report the statistical significance of the results.

Ethical concerns

This study was approved by the College of Medicine Institutional Review Board; it was conducted at King Khalid University Hospital and King Saud University Female Campus. Voluntary participation was insured by requiring all participants to provide informed consent. The consent form was clear, indicating the purpose of the study and the right of the participant to withdraw at any time without any obligation toward the study team. Participants’ anonymity was ensured by assigning each participant a code number for the purpose of analysis only. No incentives or rewards were given to participants. There was no conflict of interest to be reported.

## Results

A total of 414 students met the inclusion criteria; most of them were of Saudi nationality. The participants were university students in Riyadh; 280 of them were medical students and 134 studied in non-medical colleges. Of the 414 students, 15 (3.6%) were married. The mean length of menstruation was 6.5 days.

The majority of the study subjects (59.4%) suffered from moderate PMS while 32.6% and 8% of them suffered from mild and severe PMS, respectively. Most participants obtained normal scores in the DASS criteria: 80.9% for anxiety, 74% for depression, and 88.2% for stress. Notably, stress was the least prevalent among the participants. Depression was found to be the most prevalent with a mild score of 12.3% and a severe score of 7% (Table 1).

**Table 1:** Severity of PMS and prevalence of anxiety, depression, and stress PMS: premenstrual syndrome

	N (%)
Severity of PMS	
Mild	135 (32.6)
Moderate	246 (59.4)
Severe	33 (8)
Anxiety	
Normal	335 (80.9)
Mild	32 (7.7)
Moderate	37 (8.9)
Severe	7 (1.7)
Extremely Severe	3 (0.7)
Depression	
Normal	307 (74)
Mild	51 (12.3)
Moderate	49 (11.8)
Severe	7 (1.7)
Stress	
Normal	365 (88.2)
Mild	35 (8.5)
Moderate	14 (3.4)

To compare the mean values of PMS scores and anxiety, depression, and stress scores, an independent-samples t-test was performed. Depression scores significantly varied between medical and non-medical students at p = 0.033. Similarly, the difference between the stress scores of the two groups was significant at p = 0.011. Meanwhile, PMS and anxiety scores were similar in both groups, with no statistically significant difference. See Table 2.

**Table 2:** Comparison of mean values of PMS score and anxiety, depression, and stress scores between students of medical and non-medical colleges PMS: premenstrual syndrome

Outcome Variable	Medical	Non-Medical	t-value	p-value	95% CI
PMS Score	28.00	28.83	-0.708	0.479	(-3.115,1.465)
Anxiety Score	3.9214	4.4030	-1.103	0.271	(-1.33986, 0.37675)
Depression Score	5.8857	7.1269	-2.133	0.033	(-2.38474, -0.09756)
Stress Score	6.9143	8.2901	-2.549	0.011	(-2.43853, -0.31499)

The results shown in Table 3 indicate a strong correlation between PMS and anxiety, depression, and stress. The “r” value was measured for each of them. Stress was found to have the highest correlation with PMS while depression has the lowest; anxiety was found to be somewhere in between. In 39.8% of the participants, PMS was found to be due to stress, whereas in 33.7%, PMS was due to anxiety. Meanwhile, 29.8% of the participants had PMS that was due to depression. The correlation coefficients between PMS and all disorders were strongly significant (< 0.0001).

**Table 3:** Correlation between PMS score and anxiety, depression, and stress scores PMS: premenstrual syndrome

	PMS (“r”)	("r^2^”)	p-value
Anxiety	0.581	33.7%	<0.0001
Depression	0.546	29.8%	<0.0001
Stress	0.631	39.8%	<0.0001

As regards PMS management, the results presented in Table 4 indicate a slight distinction between medical and non-medical students. Both groups widely used over-the-counter medications (53.2%) and alternative therapy (76.8%) while visiting psychiatric clinics (0.4%) and gynecological clinics (15.6%) was less prevalent. Medical students, however, are more likely than non-medical students to seek gynecological therapy. Visiting psychiatric clinics was the least common type of management in both groups while the most common type of management is using alternative therapy. Among medications, painkillers dominate as prescribed medication (13.4%) and as over-the-counter drugs (51.8%). In both groups, the most commonly used painkillers are acetaminophens (23.3%), and the least commonly used painkillers are oxicam derivatives (0.2%). The usage of non-steroidal anti-inflammatory drugs (NSAIDs) is 11% higher among medical students while the usage of acetaminophens is 6% higher among non-medical students. The effectiveness scale found that medical students’ choices of PMS management methods are as effective as non-medical students.’ In both groups, only 12.5% reported their management method as “ineffective” while 29.6% reported it as “very effective,” 38% reported it as “effective,” and 17.5% reported it as “somewhat effective.”

**Table 4:** Management of PMS among medical and non-medical students PMS: premenstrual syndrome

Type of Management	Medical N (%)	Non-medical N (%)	Total N (%)
Psychiatric	1 (0.35)	1 (0.74)	2 (0.48)
Antidepressant Medication	1 (0.35)	0 (0.00)	1 (0.24)
Psychotherapy	0 (0.00)	1 (0.740)	1 (0.24)
Gynecological	45 (16.07)	20 (14.81)	65 (15.66)
Contraceptive Pills	2 (0.71)	0 (0.00)	2 (0.48)
Painkillers	41 (14.64)	15 (11.11)	56 (13.49)
Other	2 (0.71)	5 (3.70)	7 (1.68)
Over-the-Counter Drugs	156 (55.71)	65 (48.14)	221 (53.25)
Painkillers	154 (55.00)	61 (45.18)	215 (51.80)
Other	2 (0.71)	4 (2.96)	6 (1.44)
Alternative Therapy	219 (78.21)	100 (74.07)	319 (76.86)

## Discussion

This study measured the prevalence of PMS and its various levels, examined the methods used in its management, and investigated its correlation with anxiety, depression, and stress. Using the ACOG criteria, the majority of participants (59.4%) reported having moderate PMS while only 8% had severe PMS. This finding aligns with a study conducted among Turkish medical students that found 47% and 5.8% to have moderate and severe PMS, respectively [1]. Another study in Saudi Arabia and Iran found similar results [6,11]. An Indian study also found that 18.4% of respondents have moderate to severe PMS, and 3.7% have premenstrual dysphoric disorder [12].

To relieve their symptoms, medical and non-medical students sought similar methods, with 16.07% of medical students seeking gynecological care as compared to 14.81% of non-medical students. The use of over-the-counter medications by 7% between the two groups, with higher use among medical students (55.71%). The use of painkillers was higher among medical students at 55% while it was at 45.18% among non-medical students. These results align with our hypothesis that the use of medication to relieve PMS is higher in medical students than it is in non-medical students. The most prevalent method in PMS management in both groups was the use of alternative therapy, with 78.21% and 74.07% among medical and non-medical students, respectively. This method includes the use of heat pads and hot drinks as well as dietary changes and rest. Notably, a study reviewed the management of PMS and found that 48% of students in the College of Health Sciences at Mekelle University, Ethiopia, sought medical treatment for their symptoms. Of these students, 36.4% used painkillers while 7.5% used hot drinks [13]. A study was also conducted at a Thai high school and found that 38.4% used analgesic drugs to cope with PMS, whereas 26.3% coped by engaging in recreational activities [14]. In 2016, a Turkish article reported that 40% of health science students used oral medications while 39.1% and 33.6% used warm bags and rest, respectively [15]. In accordance with our results, the literature showed a conspicuous variation in the methods of management of PMS, which depicts the lack of consensus of PMS management. This calls attention to the need for specialized clinics that can counsel females accordingly.

In a population-based sample study that examined the relationship between PMS and depression in women of reproductive age, the prevalence of major depression was 11.3% in women with moderate PMS and 24.6% in women with severe PMS [16]. In our study, when assessing the levels of anxiety, depression, and stress using DASS. Depression and stress scores varied significantly between medical and non-medical students, with the latter having higher mean scores. Non-medical students had mean scores of 7.12 and 8.29 for depression and stress, respectively, as compared to the 5.88 and 6.91 of medical students. Al Ahsa’s study also measured the prevalence of depression, anxiety, and stress using DASS [6]. Among students diagnosed with PMS, the prevalence of depression is 27%, anxiety is 40.4%, and stress is 31.5%.

Most previous papers focus on the prevalence of PMS while we provided an insight into its association with depression, anxiety, and stress as well. In this study, we found a positive correlation between PMS and depression, anxiety, and stress. The highest correlation was between PMS and stress with an r value of 0.631. The correlation with anxiety came second with an r value of 0.581 and the lowest correlation was with depression with an r value of 0.546. This further emphasizes the implications of this phenomenon and the importance of addressing it as medical professionals.

Conversely, one of the major limitations we encountered in this study was the lack of response from participants. This might be because the questionnaire was sent via email. Hence, future studies should employ either interviews or paper-based questionnaires. We also found a recall bias since the ACOG criteria recommend that the questionnaire be filled over three consecutive months. To avoid this, we recommend that future studies should be prospective in nature, with three questionnaires filled out over three upcoming months.

## Conclusions

In conclusion, although many females suffer from PMS, it is not thoroughly addressed as a problem that might interfere with women’s physical and psychological health. The results of the current study show that the majority of women manage their PMS symptoms without guidance or consultation, which proposes the possible benefits that specialized PMS clinics can offer females. Moreover, PMS should be thoroughly studied, focusing on raising public awareness and establishing clear guidelines and regulations on dispensing pharmaceutical drugs used in the management of PMS symptoms.
